# Childhood-onset primary Sjögren’s Syndrome presenting as nephrotic syndrome: a case report and literature review

**DOI:** 10.1186/s12887-025-06029-1

**Published:** 2025-10-02

**Authors:** Yuanjin Song, Lili Sun, Guangmei Cui, Dongning Feng, Qing Sun, Yibing Wang

**Affiliations:** Department of Nephrology and Immunology, Qingdao Women and Children’s Hospital, Qingdao, China

**Keywords:** Primary Sjögren’s Syndrome, Nephrotic Syndrome, Membranous Nephropathy, Children

## Abstract

**Background:**

Pediatric primary Sjögren's syndrome typically presents with oral and ocular dryness, along with a broad spectrum of extraglandular manifestations affecting multiple organ systems. Among renal manifestations, tubulointerstitial nephritis is most commonly observed, whereas glomerular involvement is exceedingly rare.

**Case presentation:**

We report the case of an 8-year-old girl referred for evaluation of persistent foamy urine. Laboratory investigations revealed significant proteinuria and hypoalbuminemia. Kidney biopsy confirmed membranous nephropathy. Further evaluation indicated ocular involvement, evidenced by positive Schirmer's I test and reduced tear film break-up time. A labial salivary gland biopsy demonstrated focal lymphocytic infiltration. The patient was diagnosed with primary Sjögren's syndrome and was treated with corticosteroids and immunosuppressive agents, resulting in a favorable outcome and remission of proteinuria.

**Conclusions:**

This case underscores the diverse clinical spectrum of primary Sjögren's syndrome and highlights the potential for rare glomerular involvement in children. It emphasizes the need for heightened awareness among pediatric healthcare providers regarding the systemic manifestations of primary Sjögren's syndrome to prevent delayed diagnosis.

## Background

Primary Sjögren’s syndrome (pSS) is a chronic autoimmune disorder characterized by B-cell hyperactivity and lymphocytic infiltration of exocrine glands, primarily the salivary and lacrimal glands [1]. Pediatric-onset pSS accounts for approximately 1% of all cases, is characterized by high heterogeneity and frequently presents with non-specific symptoms such as oral and ocular dryness, along with a wide range of extraglandular manifestations [1–7].

Kidney involvement in pediatric pSS has been reported in approximately 5% to 20.5% of cases, with tubulointerstitial nephritis (TIN) being the most common renal manifestation [2–8]. Clinically, TIN may present as renal tubular acidosis (RTA), hematuria, glycosuria, hypokalemia, Fanconi syndrome, diabetes insipidus, and other forms of tubular dysfunction [2–8]. While glomerular lesions are not uncommon in adult pSS, they are exceedingly rare in the pediatric population [9–20]. Here, we report a pediatric case of pSS that initially presenting as nephrotic syndrome, and provide a review of the existing literature with a particular focus on glomerular involvement. To date, only fourteen biopsy-confirmed cases of glomerular disease in pediatric pSS have been documented. This case, along with insights from the literature, underscores the heterogeneous clinical presentations of pediatric pSS and aims to raise awareness among pediatric practitioners to facilitate earlier recognition and diagnosis.

## Case presentation

In September 2024, an 8-year-old girl presented to our hospital with a one-month history of persistent foamy urine. Three days prior to her admission, she developed periorbital edema. She denied experiencing any dryness in the skin, mouth, or eyes, and reported no fever, cough, rash, oral ulcers, dental caries, conjunctivitis, lymphadenopathy, or arthritis.Her vital signs were stable, with a blood pressure of 102/66 mmHg. Physical examination was largely unremarkable, except for periorbital and bilateral lower extremity edema. She weighed 24 kg and was 128 cm tall, corresponding to a body mass index (BMI) of 14.6 kg/m^2^. The patient was born at a gestational age of 38 weeks to non-consanguineous parents and had no significant family history of kidney disease or autoimmune disorders. Her growth parameters and psychomotor development were appropriate for her age.

Laboratory investigations demonstrated severe proteinuria, evidenced by a urine albumin dipstick result of 4 + and a 24-h urinary protein excretion of 6906 mg (287.8 mg/kg; reference range: 0–150). Notable biochemical abnormalities included hypoalbuminemia (albumin: 19.3 g/L; reference range: 39–54 g/L), hypercholesterolemia (cholesterol: 9.7 mmol/L; reference range: < 5.2 mmol/L), and mild electrolyte disturbances characterized by hypokalemia (potassium: 3.3 mmol/L; reference range: 3.7–5.2 mmol/L) and hyponatremia (sodium: 131 mmol/L; reference range: 135–145 mmol/L). The coagulation profile showed reduced antithrombin III activity at 34.5% (reference range: 75.0–125.0%). Elevated inflammatory markers were noted, including an erythrocyte sedimentation rate of 64 mm/hour (reference range: 0–20) and ferritin levels of 551.0 ng/mL (reference range: 13–150). The complete blood count, kidney function tests, liver function tests, C-reactive protein, antistreptolysin O, and thyroid function tests all remained within normal ranges. Serological screenings for HIV, HBV, and HCV were negative, and no bacterial growth was observed in the urine culture. Immunological evaluation revealed a positive antinuclear antibody (ANA) with a speckled pattern at a titer of 1:320. The extractable nuclear antigen panel showed strong positivity for anti-SSA antibodies, with anti-Ro-52 and anti-Ro-60 antibodies at 154 and 143 IU/mL, respectively, as well as positive anti-SSB antibodies at 59 IU/mL. Both anti-dsDNA and anti-neutrophil cytoplasmic antibody (ANCA) tests were negative. Serum immunoglobulin analysis revealed an elevated IgE level of 455.0 IU/mL (reference range: 0–90), normal IgG levels of 12.5 g/L (reference range: 6.3–15.0), and elevated IgA and IgM levels of 2.97 g/L (reference range: 0.46–2.51) and 2.79 g/L (reference range: 0.47–2.20), respectively. Complement components C3 and C4 were within the normal range. Serum phospholipase A2 receptor (PLA2R) antibody testing was negative.Imaging studies revealed bilateral enlargement of the parotid gland on ultrasonography. A chest computed tomography scan demonstrated pneumonia accompanied by bilateral pleural effusion and partial compressive atelectasis of the left lower lobe. Ultrasonography of the urinary system, electrocardiogram, and echocardiography findings were unremarkable.

**Fig. 1:**
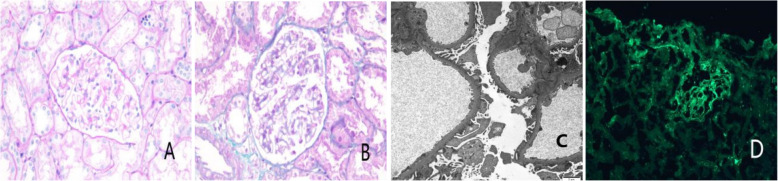
Histology of the Patient’s Kidney Biopsy. **A** and **B** Light microscopy shows mild segmental proliferation in the mesangial cells and matrix with basement membrane thickening evident in PAS (**A**) and Masson’s trichrome (**B**) stains. **C** Electron microscopy reveals segmental basement membrane thickening and extensive foot process effacement. **D** Immunofluorescence demonstrates moderate granular IgG deposits (+ +) along capillary loops. PAS, Periodic acid–Schiff

Given the clinical presentation suggestive of secondary nephrotic syndrome, a kidney biopsy was performed, which confirmed stage I membranous nephropathy (MN) (Fig. 1). Light microscopy revealed 18 glomeruli with no evidence of global or segmental sclerosis. There was mild segmental proliferation of mesangial cells and matrix, along with thickening of the glomerular basement membrane and focal vacuolar changes. Tubular epithelial cells exhibited granular and vacuolar degeneration without significant atrophy. Electron microscopy showed segmental thickening of the basement membrane with extensive foot process effacement and scattered subepithelial electron-dense deposits, occasionally extending into mesangial areas. Immunofluorescence demonstrated granular IgG deposition (2 +) along the capillary loops, with trace staining for IgA ( ±) and C1q ( ±), focal IgM deposition (1 +), and negative staining for C3.

Suspecting SS, the child underwent ophthalmological tests, which showed positive results for both Schirmer's I test and the tear film break-up time examination. A labial salivary gland biopsy demonstrated focal lymphocytic infiltration in the interstitium, with 1–6 foci per 4 mm^2^ (each focus containing more than 50 lymphocytes). The calculated focus score was ≥ 1 focus per 4 mm^2^.

The patient was diagnosed with pSS and initially treated with daily oral prednisone (2 mg/kg), anticoagulation therapy using low-molecular-weight heparin, and supportive care. After nearly two weeks of treatment, urinalysis continued to show significant proteinuria. As a result, pulse therapy with intravenous methylprednisolone (15 mg/kg/day for three days) was initiated, followed by continued oral prednisone (2 mg/kg/day) and monthly intravenous cyclophosphamide (0.5 g/m^2^). This regimen led to the normalization of the patient's urinary protein levels. One month later, the prednisone dose was gradually tapered, and after two months, cyclophosphamide was replaced with daily mycophenolate mofetil at a dose of 25 mg/kg. At the most recent follow-up, eight months after discharge, the patient’s urinalysis was normal, and the urine protein-to-creatinine ratio (UPCR) was 0.19 mg/mg (reference range: < 0.2), with no other clinical signs or symptoms of pSS. However, immunological testing continued to reveal an elevated ANA titer of 1:320, along with persistent positivity for anti-SSA and anti-SSB antibodies.

## Discussion and conclusions

This case presents a rare and atypical clinical presentation of pediatric pSS, initially manifesting solely as nephrotic syndrome secondary to MN. The patient did not exhibit typical sicca symptoms, such as dry mouth or dry eyes. A comprehensive evaluation subsequently confirmed the diagnosis of pSS. Treatment with corticosteroids and immunosuppressive agents led to a favorable clinical response and complete remission of proteinuria. To our knowledge, this is an uncommon presentation of childhood-onset pSS, as glomerular involvement, particularly in the form of MN, is exceedingly rare in pediatric patients. This case highlights the diagnostic challenges associated with extraglandular-dominant phenotypes and broadens the clinical spectrum of renal manifestations in pediatric pSS.

A comprehensive literature search was conducted across PubMed, Web of Science, China National Knowledge Infrastructure (CNKI), and the Wanfang Database to identify reported cases of glomerular involvement in pediatric pSS published up until January 2025. The search utilized the following keywords: juvenile, pediatric, child, children, adolescent, primary Sjögren’s syndrome, pSS, glomerulopathy, glomerulonephritis, glomerular disease, membranous nephropathy, minimal change disease and kidney biopsy. This search yielded 14 biopsy-confirmed cases of glomerular disease in pediatric pSS (Table 1) [4, 9–20]. These cases encompassed a wide spectrum of histopathological patterns and clinical presentations. Reported glomerular pathologies included MN, IgA nephropathy, crescentic glomerulonephritis (GN), immune complex-mediated membranoproliferative GN, focal proliferative GN, focal segmental glomerulosclerosis (FSGS), and extensive glomerulosclerosis [4, 9–20]. TIN coexisted in 8 out of the 14 cases.This histopathological heterogeneity was associated with a broad range of clinical manifestations, ranging from mild hematuria and proteinuria to nephrotic-range proteinuria and even rapidly progressive renal failure requiring dialysis [9–20]. Given the diversity of renal pathology and the potential severity of renal involvement, kidney biopsy is strongly recommended in pSS patients presenting with signs of renal impairment, particularly when glomerular disease is suspected. Although the precise mechanisms underlying glomerular injury in pSS remain unclear, current evidence suggests that the deposition of circulating immune complexes likely plays a pathogenic role [1].

**Table 1:** Glomerular involvement in pediatric primary Sjögren’s syndrome

Reference	Gender/Age	Kidney manifestations	Kidney biopsy	Treatment
Yoshida et al. (1996) [9]	M/13	Hematuria and proteinuria 24-h proteinuria of 0.3 g	MN	Mizoribin,GC
Pessler et al. (2006) [10]	F/10	Polyuria, polydipsia hyposthenuria	Extensive glomerulo-sclerosis, TIN	GC,CTX,MMF
Johnson et al.(2007) [11]	M/13	Heavy proteinuria Mild hematuria End-stage renal failure	Extensive glomerular sclerosis, TIN	GC (Pulse GC) AZA Haemodialysis
Johnson et al. (2007) [11]	M/14	End-stage renal failure	Extensive glomerular sclerosis, TIN	CVVH Haemodialysis
Jung et al. (2010) [12]	M/11	Gross hematuria and mild proteinuria	IgAN	GC
Ito et al. (2010) [13]	F/10	Proteinuria,microscopic hematuria	IgAN	Low dose GC
Ito et al. (2010) [13]	F/12	Proteinuria, microscopic hematuria	IgAN	None
Kagan et al.(2011) [14]	F/12	Moderate proteinuria, microscopic hematuria, acute renal failure	Crescentic GN TIN	GC (Pulse GC) PE, CTX,AZA
Kobayashia et al. (2020) [15]	F/11	Proteinuria, haematuria, acute renal failure	APSGN TIN	GC,penicillinG
Pehlivanoglu et al. (2021) [16]	F/16	Heavy proteinuria, microscopic hematuria, decreased GFR	IC-MPGN	GC (Pulse GC) HCQ, MMF rituximab
Mishra et al. (2023) [17, 18]	F/9	Nephrotic syndrome	FSGS,mild TIN	GC (Pulse GC) HCQ,TAC
Gong et al. (2023) [4]	NM	Proteinuria	Focal proliferative GN with mild TIN	NM
Şenol et al. (2023) [19]	F/16	Proteinuria, 24-h urine protein excretion was 1350 mg/day	FSGS with mild TIN	GC,HCQ
Bouchalova et al. (2024) [20]	M/ 15.5	Sterile pyuria, elevated albumin/creatinine ratios, mild erythrocyturia, slightly decreased glomerular filtration	NM	GC,MP pulse HCQ
Our case	F/8	Nephrotic syndrome	MN	GC (Pulse GC) CTX, MMF

*MN* membranous nephropathy, *GC* glucocorticoid, *TIN* tubulointerstitial nephritis, *CTX* cyclophosphamide, *MMF* mycophenolate mofetil, *Pulse GC* Glucocorticoid Pulse Therapy, *AZA* azathioprine, *CVVH* continuous venovenous haemofiltration, *IgAN* IgA nephropathy, *GN* glomerulonephritis, *PE* plasma exchange, *APSGN* Acute Post Streptococcal Glomerulonephritis, *GFR* glomerular filtration rate, *IC-MPGN* Immune complex-mediated membranoproliferative glomerulonephritis, *HCQ* Hydroxychloroquine, *FSGS* focal segmental glomerulosclerosis, *TAC* tacrolimus, *NM* not mentioned

Currently, no established guidelines exist for the management of glomerular disease associated with pSS [8, 21, 22]. Therapeutic strategies are therefore individualized, primarily guided by the patient’s pathological findings and clinical experience, with glucocorticoids and immunosuppressive agents as the main therapeutic approaches [8, 21, 22]. However, careful long-term follow-up is essential to monitor disease progression and manage potential relapses or complications.

Glomerular involvement is more frequently reported in adults with pSS. In fact, MN is considered one of the most frequently observed types of glomerular lesions in adult pSS patients. Recent studies have shown that MN accounts for approximately 36% to 50% of renal lesions in this population [23–25]. Despite its frequency in adults, MN remains exceedingly rare in pediatric pSS, with only two biopsy-confirmed cases reported to date, including the present case. This contrast underscores the importance of recognizing atypical renal manifestations in children and maintaining long-term renal surveillance in pediatric pSS patients.

To date, there are no universally validated diagnostic criteria for childhood-onset SS. The American-European Consensus Group (AECG) criteria and the American College of Rheumatology (ACR)/EULAR classification criteria were initially developed for adult populations and have not yet been sufficiently validated in pediatric settings [4, 7, 26–29]. In the current case, diagnosis was based on the 1999 diagnostic criteria for juvenile pSS and the pediatric guidelines commonly used in Japan [28, 29]. However, even these pediatric-specific criteria require further validation, underscoring the need for additional research in this field [4, 30, 31]. Furthermore, existing pediatric diagnostic criteria may not sufficiently address certain extraglandular manifestations, particularly renal involvement such as glomerular disease, potentially leading to underdiagnosis or delayed diagnosis in affected children.

This report presents a rare case of pSS in a pediatric patient with nephrotic syndrome as the initial manifestation. For children presenting with nephrotic syndrome, comprehensive immunological screening is essential to establish an accurate diagnosis. Additionally, children with pSS should undergo regular, long-term kidney assessments, regardless of whether renal involvement is present at the time of diagnosis. Early recognition and intervention are key to preventing long-term complications and improving clinical outcomes.

## Data Availability

No datasets were generated or analysed during the current study.

## Abbreviations

pSS: Primary Sjögren’s syndrome
TIN: Tubulointerstitial nephritis
RTA: Renal tubular acidosis
BMI: Body mass index
ANA: Antinuclear antibody
PLA2R: Phospholipase A2 receptor
ANCA: Anti-neutrophil cytoplasmic antibody
MN: Membranous nephropathy
UPCR: Urine protein-to-creatinine ratio
CNKI: China National Knowledge Infrastructure
GN: Glomerulonephritis
IC-MPGN: Immune complex-mediated membranoproliferative glomerulonephritis
FSGS: Focal segmental glomerulosclerosis
AECG: American-European Consensus Group
ACR: American College of Rheumatology
